# Revisiting down syndrome through the lens of interferonopathy and innate immune dysregulation

**DOI:** 10.3389/fimmu.2026.1836558

**Published:** 2026-06-03

**Authors:** Guangfu Wang, Weili Shi, Shixiu Liao, Bingtao Hao

**Affiliations:** 1 Medical Genetic Institute of Henan Province, Henan Key Laboratory of Genetic Diseases and Functional Genomics, People’s Hospital of Zhengzhou University, Zhengzhou University, Zhengzhou, China; 2 Department of Immunology, School of Basic Medical Sciences, Zhengzhou University, Zhengzhou, China

**Keywords:** AP-1, autoinflammation, chromatin accessibility, cytokines, Down syndrome, interferonopathy, JAK-STAT, m6A epitranscriptomics

## Abstract

Down syndrome (DS), caused by trisomy 21, has long been viewed primarily as a neurodevelopmental disorder. However, increasing evidence indicates that it is also associated with pervasive immune dysregulation, including chronic inflammation and heightened susceptibility to autoimmunity. Here, we revisit DS from the perspective of innate immunity and suggest that it shares key features with interferon-driven, autoinflammation-like conditions. Drawing on recent multi-omics studies, we outline a mechanistic framework linking chromosome 21 gene dosage to systemic immune activation. Increased expression of interferon receptors lowers the threshold for signaling and drives persistent activation of interferon-stimulated genes (ISGs). In parallel, reduced METTL3-dependent m^6^A modification may stabilize pro-inflammatory transcripts and enhance innate immune sensing. These changes occur alongside chromatin accessibility remodeling enriched for AP-1–associated elements, consistent with a transcriptionally primed state that amplifies inflammatory gene expression. Together, these processes form a feed-forward network involving interferon signaling, transcriptional activation, and cytokine production, providing a basis for the basal inflammatory state in DS and its high burden of immune-mediated comorbidities. This framework also highlights potential therapeutic opportunities, including JAK–STAT inhibition and cytokine-targeted approaches, which may help restore immune homeostasis and inform future translational studies.

## Graphical Abstract

**Figure:**
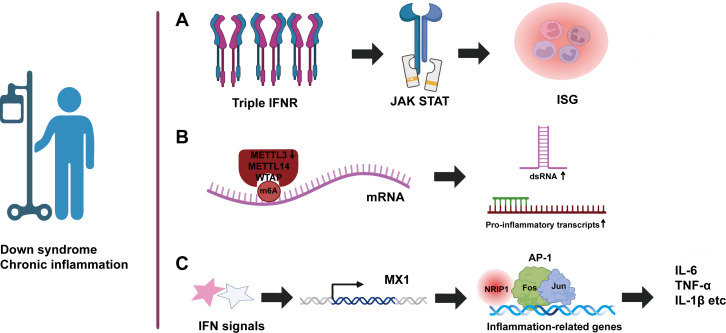
Trisomy 21 promotes chronic inflammatory priming through interferon receptor gene dosage, m^6^A dysregulation, and AP-1–associated chromatin remodeling. **(A)** In trisomy 21, increased dosage of chromosome 21–encoded interferon receptor genes, including *IFNAR1*, *IFNAR2*, *IFNGR2*, and *IL10RB*, lowers the threshold for interferon responsiveness and promotes chronic JAK–STAT activation. This results in sustained expression of interferon-stimulated genes, including *MX1*, and contributes to basal inflammatory activation. **(B)** Reduced *METTL3* expression compromises the activity of the METTL3/METTL14/WTAP m^6^A writer complex, leading to decreased m^6^A modification on selected transcripts. Loss of m^6^A-dependent RNA regulation may impair normal RNA turnover, stabilize specific pro-inflammatory transcripts, and promote accumulation of endogenous double-stranded RNA species, thereby enhancing innate immune sensing. **(C)** Elevated *MX1* and persistent interferon signaling amplify AP-1–associated transcriptional programs, including *FOS* and *JUN*. AP-1 activity promotes chromatin accessibility at inflammatory regulatory elements and drives expression of pro-inflammatory cytokines such as IL-6, TNF-α, and IL-1β. Together, these interconnected mechanisms establish a chronic “pre-activated” inflammatory state in Down syndrome cells.

## 1 Introduction

DS is a common genetic condition caused by an extra copy of chromosome 21 (trisomy 21) (1, 2). Beyond the well-known cognitive and developmental manifestations, individuals with DS suffer a substantial medical burden from immune dysfunction (3). DS confers markedly elevated susceptibility to infections and a broad spectrum of autoimmune diseases (4, 5). For example, people with DS are prone to recurrent respiratory infections and severe viral pneumonias, and exhibit high frequencies of autoimmune thyroiditis, type 1 diabetes, celiac disease, alopecia areata, and arthritis compared to the general population (6–9). In one study, up to 22 inflammatory cytokines were found to be persistently elevated in the plasma of adults with DS even in the absence of infection, reaching levels that exceeded those seen in acutely ill patients (8, 10). Immune cell profiling reveals basal activation of multiple leukocyte subsets: DS individuals show chronic IL-6 signaling in CD4^+^ T cells and expansion of CD11c^+^ T-bet^high^ atypical B cells that are poised for autoantibody production (8, 11, 12). Consistently, hundreds of autoantibodies targeting diverse tissues (thyroid, pancreas, gastrointestinal tract, central nervous system, etc.) can be detected in the blood of adults with DS (8, 12, 13). These findings point to an autoimmunity-prone, hyperinflammatory baseline state in DS.

Crucially, many features of DS immune dysregulation resemble those of systemic autoinflammatory disorders. Autoinflammatory diseases are characterized by innate immune activation, cytokine overproduction, and inflammation in the absence of specific autoantigens (11, 14). In DS, innate immune pathways appear chronically “turned on” from early life: interferon-stimulated gene expression is upregulated in multiple cell types, and inflammatory cytokines like IL-1, IL-6 and TNF are basally elevated (8, 15, 16). These immune perturbations occur even before overt autoimmune disease develops (3), suggesting a primary innate immune imbalance. The high prevalence of autoimmune conditions in DS (e.g. celiac disease affecting ~5% (17, 18) and thyroid disease up to ~18% (17) of individuals) further indicates immune system misregulation. Given these findings, there is a growing rationale to view DS through the lens of immune pathogenesis and classify it as an autoinflammatory disease (11, 19). In the sections below, we synthesize multi-omic data illuminating the mechanisms by which trisomy 21 causes systemic inflammation, and we highlight therapeutic avenues targeting the innate immune axis in DS (**Table 1**).

**Table 1:** Changes of inflammatory mediators in Down syndrome.

Cytokine	Sample source	Direction of change	Reference
IL-6, MCP-1, IL-22, TNF-α, B2M, VEGF-A, TFF3	peripheral plasma	upregulated	(20)
IL-6, IL-1α, TNFβ, IL-4, IL-13, IL-12, IFNγ, IL-17A, IL-2, IL-10, IFNα2	peripheral plasma	upregulated	(8)
TNFα, IL-6, TSLP, CXCL10, CRP, SAA, MIP-1, MIP-3α, VEGF-C,	peripheral plasma	upregulated	(21)
TNF-α, IL-1β, IFN-γ, Neopterin	peripheral plasma	upregulated	(15)
CRP, SAA, IL-1RA, TSLP, IL-17C, IL-17D, IL-22, IL-9, IL-6, TNF-α, IP-10, MIP-3α, MIP-1α, MCP-4, PIGF, VEGF-A	peripheral plasma	upregulated	(3)

## 2 Epitranscriptomic dysregulation: METTL3/m^6^A and inflammatory consequences

**Figure 1:**
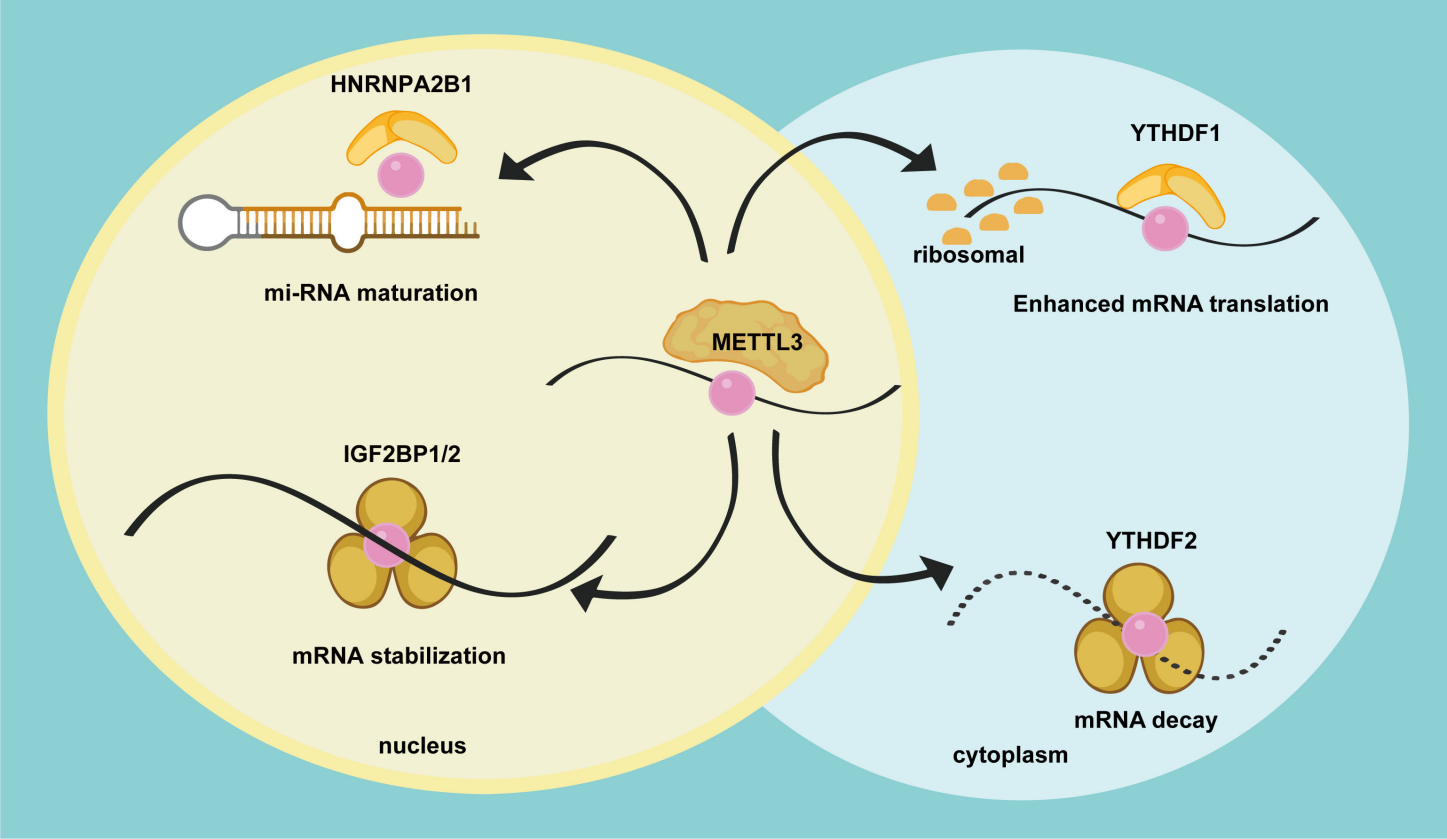
Epitranscriptomic regulation of RNA fate *via* METTL3 and m^6^A readers (27). METTL3 catalyzes the deposition of m^6^A marks (pink dots) on mRNAs. In the nucleus, m^6^A is recognized by HNRNPA2B1, which promotes microRNA processing, and by IGF2BP1/2, which enhance mRNA stability. In the cytoplasm, YTHDF1 promotes efficient translation, whereas YTHDF2 mediates mRNA decay. Thus, the fate of m^6^A-modified transcripts is determined by the specific reader proteins that bind them, regulating either mRNA stability or degradation.

Epitranscriptomic modifications of RNA, particularly N^6^-methyladenosine (m^6^A), have recently been recognized as critical regulators of gene expression and immune homeostasis (22–25). M^6^A marks on messenger RNA influence transcript splicing, stability, and translation by recruiting specific “reader” proteins. METTL3 is the catalytic subunit of the m^6^A methyltransferase complex, encoded by the *METTL3* gene located on chromosome 14 (14q11.2). The methyltransferase METTL3 catalyzes m^6^A installation, working in a complex with METTL14 and WTAP (22). Notably, METTL3 activity can both promote and restrain inflammation in different contexts by modulating the lifespan of mRNAs that encode cytokines or signaling molecules (22, 26). In DS, emerging evidence indicates a global disruption of m^6^A epitranscriptomic marks that may contribute to aberrant inflammatory signaling (**Figure 1**).

In the context of DS, recent work has shown that m^6^A modifications are reduced genome-wide in DS tissues, likely due to deficient METTL3 activity (28). Our study of human fetal DS brain cortex revealed significantly lower m^6^A levels on mRNAs compared to euploid controls, accompanied by a reduction in METTL3 protein expression in DS samples (28, 29). Consistent with a loss of m^6^A “writer” function, hundreds of genes in DS brain exhibited abnormally increased transcript levels along with decreased m^6^A marks (28). One example is the nuclear receptor co-regulator NRIP1 (RIP140), encoded on chromosome 21, which showed a 3–5 fold increase in mRNA and protein expression in DS brains while its m^6^A modification was reduced by 2–3 fold (28). Experimental manipulation of METTL3 in cell models confirmed that NRIP1 is a direct m^6^A-regulated transcript: METTL3 knockdown led to loss of m^6^A on *NRIP1* mRNA and a corresponding stabilization and accumulation of the transcript (30). Conversely, METTL3 overabundance increased m^6^A on *NRIP1* and accelerated its mRNA decay. Thus, in DS, insufficient METTL3/m^6^A likely prolongs the lifespan of specific mRNAs, leading to the overabundance of genes that can disrupt cellular metabolism and immune balance.

Notably, *NRIP1* overabundance has been linked to mitochondrial dysfunction in DS neurons (28), which could have broader inflammatory consequences. Impaired mitochondrial function can trigger innate immune pathways (e.g. *via* reactive oxygen species and inflammasome activation) (31, 32). More generally, the epitranscriptomic dysregulation in DS may affect immune cell gene expression programs. For instance, if m^6^A is broadly decreased in DS immune cells (as seen in brain tissue), transcripts encoding negative regulators of inflammation might be stabilized inappropriately. Alternatively, pro-inflammatory mRNAs might escape normal decay. In other conditions, loss of *METTL3* in regulatory T cells was shown to stabilize SOCS family mRNAs and inhibit cytokine signaling (22, 33, 34), whereas *METTL3* deletion in dendritic cells impaired their maturation and IL-2 production (22). In DS, the net impact of m^6^A dysregulation on the immune system remains to be fully elucidated, but the data so far suggest a trend toward excess accumulation of certain chromosome 21-encoded transcripts that could perturb immune homeostasis. Restoring epitranscriptomic balance might therefore ameliorate some inflammatory aspects of DS.

**Figure 2:**
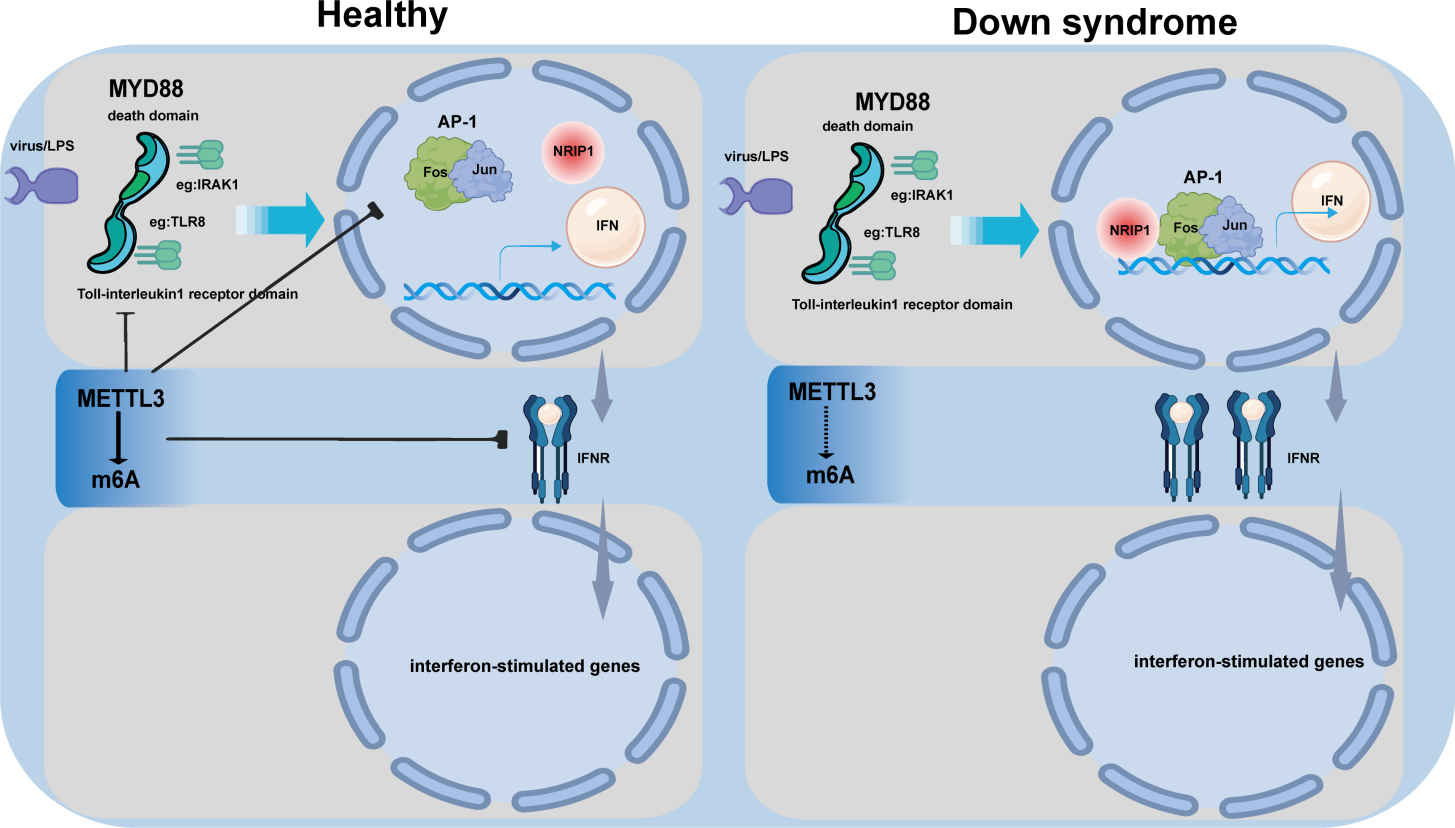
Interferon hyperactivation through AP-1 and NRIP1 in Down syndrome. In healthy individuals, METTL3-mediated m^6^A modification suppresses MYD88-dependent activation of AP-1 and NRIP1, thereby inhibiting IFN signaling and preventing excessive activation of ISGs. In contrast, in DS, this regulatory mechanism is impaired, leading to excessive inflammatory responses due to unrestrained IFN and ISG activation.

This idea is supported by the well-documented clinical presentation of DS, where individuals frequently exhibit chronic immune dysregulation and elevated systemic inflammation. While direct evidence linking METTL3-dependent m^6^A RNA methylation to these processes is still limited, emerging studies point to its likely role in the disease’s pathogenesis. There are two main mechanisms involved. First, trisomy 21 may change mRNA stability of specific genes (e.g., *SH3BGR*) *(*35) through the METTL3-m^6^A axis, potentially contributing to developmental anomalies. A broader and potentially more consequential effect relates to the role of METTL3 in maintaining cellular homeostasis by depositing m^6^A marks on transcripts derived from repetitive genomic elements (36–38), such as transposable elements (39). These modifications normally limit the accumulation of endogenous nucleic acids. Loss of *METTL3* leads to cytosolic enrichment of these endogenous nucleic acids, which are recognized by pattern recognition receptors (e.g., *TLR3*, MDA5 (40–44)), triggering aberrant innate immune responses, including JAK-STAT (45–47) signaling and NLRP3 inflammasome activation (48). In immune cells, such as γδT cells (49), this dysregulation influences inflammatory outcomes (50). In summary, impaired METTL3-mediated m^6^A modification provides a plausible molecular link between chromosome 21 trisomy and diverse clinical manifestations of DS, including congenital heart defects and immune dysregulation. These observations highlight epitranscriptomic dysregulation as an underappreciated layer of gene control in trisomy 21, with direct relevance to inflammation-driven pathology (**Figure 2**).

## 3 Chromatin accessibility and AP-1 primed inflammation in DS

Beyond transcriptome and epitranscriptome changes, trisomy 21 also alters the chromatin landscape, priming certain inflammatory genes for heightened expression (51). Chromatin accessibility profiling (ATAC-seq) of DS versus control cells reveals widespread remodeling of regulatory DNA, including gains in open chromatin at promoters/enhancers of inflammation-related genes (52). In particular, motif enrichment analysis of regions with increased accessibility in DS points to over-representation of binding sites for activator protein-1 (AP-1) transcription factors (52). AP-1 is a family of dimeric transcription factors (comprising Fos and Jun proteins) that controls genes involved in stress responses, differentiation, and immunity. The major components, *FOS* (14q24.3) and *JUN* (1p32.1), are not located on chromosome 21, yet their activity is elevated in DS. Persistent AP-1 activation is a known driver of pro-inflammatory cytokine production (for example, AP-1 binds the IL6 gene promoter to up-regulate IL-6 transcription). Evidence now suggests that trisomy 21 creates a feed-forward loop of interferon signaling and AP-1–mediated chromatin priming, which may exacerbate inflammatory gene expression in DS.

Our recent multi-omics study integrated ATAC-seq and RNA-seq in DS patient-derived cells to dissect this phenomenon (52). We found that DS cells have increased chromatin accessibility near AP-1 target genes and corresponding overabundance of many AP-1 transcriptional targets compared to euploid cells. Notably, *MX1*, an interferon-inducible antiviral gene triplicated on chromosome 21, emerged as a key upstream regulator linking interferon hyperactivity to AP-1 activation (52). *MX1* (MxA protein) is strongly upregulated by type I interferons and was highly expressed in DS cells; *MX1* overabundance alone was sufficient to induce the expression of AP-1 family transcription factors (such as *FOS* and *JUNB*), implicating *MX1* in driving the AP-1 gene network in DS. We also observed that DS cells with high MX1 showed increased expression of AP-1-dependent inflammatory genes and signs of cellular stress. Strikingly, pharmacological inhibition of AP-1 activity reversed several DS cellular phenotypes: treating DS cells with the c-Fos/AP-1 inhibitor T-5224 significantly reduced the expression of inflammation-related genes and improved cell viability and growth characteristics. This suggests that excessive AP-1 activity contributes directly to DS pathophysiology. AP-1 likely cooperates with other factors (e.g. NF-κB) to amplify production of cytokines like IL-6, TNF-α, and IL-1β in DS, given AP-1’s known role in cytokine gene regulation.

From a chromatin perspective, trisomy 21 leads to a state of promiscuous gene activation. The extra interferon-stimulated genes (like *MX1*) on chromosome 21 continuously signal, keeping AP-1 and other transcription factors (NF-κB, EGR1, etc.) activated, thereby maintaining an open chromatin state at many immune response genes. This AP-1 driven open chromatin may cause innate immune cells (monocytes, macrophages, dendritic cells) to respond hyperactively to secondary stimuli. Thus, DS cells are in a transcriptionally “primed” state for inflammation. The interplay between interferon signaling and AP-1 mediated chromatin changes provides a mechanistic link between the gene dosage effect of chromosome 21 and the aberrant immune gene expression profiles observed in DS. Therefore, targeting transcriptional activators such as AP-1 or their upstream inducers could suppress this inappropriate primed state. In summary, chromatin accessibility analysis indicates that DS skews the epigenetic landscape toward an activated immune profile, with AP-1 as a central node of inflammatory gene dysregulation (**Figure 2**).

## 4 Interferon receptor triplication and the interferonopathy of DS

One of the most significant immune perturbations in DS arises from the triple dose expression of interferon receptor genes on chromosome 21 (53). The type I interferon receptor subunits IFNAR1 and IFNAR2 (54), the type II interferon receptor subunit IFNGR2, and the interleukin-10 receptor beta (IL-10RB, which also serves as the type III interferon receptor) are all encoded on chromosome 21 (17, 55, 56). As a consequence, individuals with DS have ~1.5-fold higher expression of these receptor proteins in many cell types, lowering the threshold for interferon signaling. This gene dosage translates into a chronic state of interferon pathway activation — essentially a mild interferonopathy. Interferonopathies are autoinflammatory conditions caused by endogenous upregulation of interferons, as seen in monogenic disorders like Aicardi–Goutières syndrome. In DS, the interferon hyperactivity is polygenic but comparably results in an elevated interferon signature that pervades multiple organ systems (**Table 2**).

**Table 2:** Interferon pathway activation markers: Euploid vs. DS (17).

Marker	Euploid	Trisomy21
TypeIIFN(>10fg/ml)	1%	12%
TypeIIIFN(T cell)	++	+++
IFN-αR1	++	+++
IFN-αR2	++	+++
IFN-γR1	++	++
IFN-γR2	++	+++
STAT1/Pstat1(Monocyte)	++	+++
STAT1/Pstat1 (T-cell)	++	+
ISGF3	++	+++
GAF	++	+++
ISGs	++	+++

Summary of interferon pathway activation markers in blood cells from euploid individuals versus DS. “+++” indicates significantly elevated levels in DS. DS shows a higher proportion of individuals with detectable circulating IFN-α, heightened IFN-γ responses in T cells, overabundance of *IFNAR1/2* and *IFNGR2*, and increased STAT1 phosphorylation and ISG expression, compared to controls.

Multiple studies have demonstrated the interferon hypersensitivity and resultant inflammation in DS. Large transcriptomic analyses of blood and tissues have found consistent upregulation of interferon-stimulated genes (ISGs) in people with DS (17). Notably, Espinosa and colleagues first identified an interferon gene expression signature in cells from individuals with DS in 2016, and subsequent work confirmed that monocytes from DS patients exhibit modular IFN activation scores intermediate between healthy controls and STAT1 gain-of-function disease patients. This interferon hyperactivity in DS is sufficient to produce clinical and immunological features overlapping with classical interferonopathies: for instance, DS patients often have lymphopenia, high basal antiviral gene levels, and signs of immunosenescence. T cells in DS show reduced thymic output and naive T-cell numbers, an activated memory phenotype with high STAT1 phosphorylation, and a bias toward terminal differentiation with increased IFN-γ production (17, 57)– changes that mirror chronic interferon exposure in other contexts. Importantly, a landmark study in a DS mouse model demonstrated causality: normalizing the copy number of the interferon receptor cluster (reducing it from three copies to two) abolished many pathological features of DS mice, including inflammatory gene overabundance, heart defects, and cognitive impairment (58). Conversely, trisomic mice with the intact interferon receptor triplication showed aberrant antiviral responses and developmental deficits, which were reversed by blocking interferon signaling. These results firmly establish that trisomy of interferon receptors drives constitutive interferon/JAK-STAT pathway activation, contributing to diverse DS phenotypes (58).

**Figure 3:**
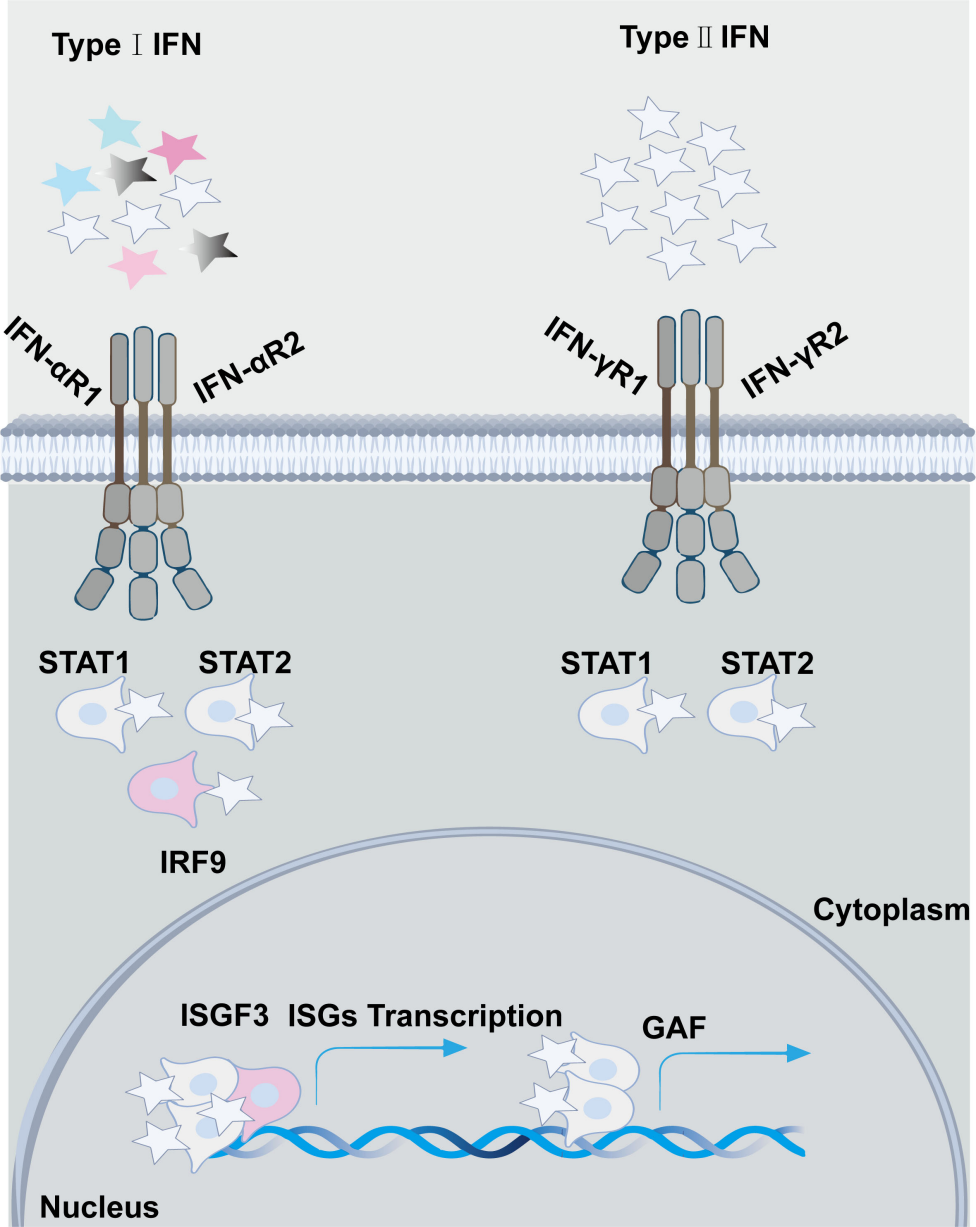
Chronic interferon hyperactivation in DS (17). Schematic of type I (IFN-α/β) and type II (IFN-γ) interferon signaling via the JAK-STAT pathway. The receptor subunits IFNAR1, IFNAR2, and IFNGR2 (in red) are triplicated in DS. Upon ligand binding, these receptors activate STAT1/STAT2, forming the ISGF3 transcription factor complex (with IRF9) for type I IFN signaling, or STAT1 homodimers (GAF) for type II IFN signaling. These drive transcription of interferon-stimulated genes (ISGs).

In human DS, the interferonopathy is considered “mild” relative to monogenic interferon-driven diseases (17)– not every individual with DS has elevated circulating interferon, and ISG levels, while higher on average, vary between patients. About 10–30% of individuals with DS may have markedly high serum type I IFNα (17, 53, 55). Even low-grade interferon signaling, however, can have insidious systemic effects over time. Interferons can inhibit hematopoiesis, skew T-cell differentiation, and induce a proinflammatory milieu. Indeed, chronic interferon signaling in DS likely underlies certain myeloid hyperplasia and autoimmunity phenomena (e.g. the high rate of hyperthyroidism and thyroid autoantibodies in DS could be related to interferon, as exogenous IFN-α therapy in euploid individuals often precipitates thyroid autoimmunity (17). The concept of DS as an interferonopathy suggests that innate immune cells are continually activated. For example, DS plasmacytoid dendritic cells produce exaggerated IFN, and DS monocytes have heightened baseline STAT1 phosphorylation and spontaneously express ISGs (17, 53, 59). This persistent interferon drive likely amplifies the inflammation from other sources (such as the chromatin/AP-1 changes described above), creating a self-reinforcing cycle of immune activation. In summary, the triplication of interferon receptor genes in DS places the condition in the spectrum of interferon-mediated autoinflammatory disorders. Recognizing DS as a type I interferon-driven syndrome provides a mechanistic framework to explain its immune dysregulation and suggests specific anti-interferon therapeutic approaches, as discussed next (**Figure 3**).

## 5 Therapeutic strategies targeting inflammation in DS

Reframing DS as a systemic autoinflammatory condition has important clinical implications. It opens the door to repurposing immunomodulatory therapies that dampen overactive innate immune pathways in DS patients. A number of targeted anti-inflammatory strategies are being explored to correct the cytokine and interferon imbalance in DS:

JAK-STAT Inhibition: Janus kinase (JAK) inhibitors, which blunt signaling downstream of interferons and many cytokines, are a promising approach in DS. A recent phase II trial of the pan-JAK inhibitor tofacitinib in young adults with DS showed reduced autoimmune skin disease activity and a normalization of inflammatory biomarkers (3, 60, 61). Treatment for 16 weeks led to improvements in alopecia areata, psoriasis, and atopic dermatitis lesions in DS patients, while significantly decreasing their interferon-responsive gene signature, lowering circulating cytokine levels, and even reducing pathogenic autoantibody titers (3, 62, 63). Notably, this immune suppression was achieved without overt immunosuppressive side effects (3). These results indicate that JAK inhibition can safely recalibrate the hyperactive immune state in DS. By blocking type I IFN signaling through IFNAR and other cytokine pathways (IL-6, IL-2, etc.), JAK inhibitors address the multifactorial inflammation in DS. Other JAK-STAT targeting drugs (e.g. baricitinib, ruxolitinib) may similarly benefit DS patients with autoimmune or autoinflammatory complications (3, 8, 20, 21). Indeed, case reports suggest JAK inhibitors can ameliorate DS-associated arthritis and even neuropsychiatric regression in some instances (3). Overall, JAK inhibitors represent a leading therapeutic candidate to treat the interferonopathy and broad immune dysregulation in DS (3, 63–68).

AP-1 Inhibition: Given the evidence that AP-1 transcription factors drive inflammatory gene expression in DS (via *MX1* and chromatin changes), targeting the AP-1 pathway is another intriguing strategy. Direct AP-1 inhibitors like T-5224 (a small molecule c-Fos/c-Jun antagonist) have shown efficacy in DS cell models (52, 69). In DS amniocyte cultures, T-5224 treatment downregulated AP-1 target genes and improved cell growth and viability, essentially rescuing some DS-associated cellular phenotypes (52). While AP-1 inhibitors are not yet in clinical use for DS, this proof-of-concept suggests that drugs dampening AP-1 activity (for example, via upstream blockade of the MAPK pathways that activate AP-1) could modulate inflammation in DS. Some existing anti-inflammatory agents have indirect anti-AP-1 effects – high-dose glucocorticoids can suppress AP-1 DNA binding, and MAPK inhibitors (like p38 inhibitors) reduce AP-1–mediated transcription. These could be explored in DS-related inflammatory conditions. Targeting AP-1 might be particularly relevant for DS patients with refractory inflammatory symptoms not fully controlled by JAK inhibitors or for tissues where local AP-1 activity (e.g. in the brain or skin) contributes to pathology.

Cytokine-Targeted Therapies: An array of biologic therapies could be repurposed to neutralize specific cytokines that are chronically elevated in DS. One of the most appealing targets is Interleukin-6 (IL-6). DS individuals often have high IL-6 levels at baseline (8, 15, 16, 70), which drive abnormal T-cell and B-cell activation (8, 71, 72). *In vitro*, blocking IL-6 signaling with the IL-6 receptor antibody tocilizumab completely normalized excessive STAT3 activation in DS immune cells (8, 57, 71). Tocilizumab is an approved therapy for other inflammatory conditions (e.g. rheumatoid arthritis) and could potentially reduce the plasmablast expansions and autoantibody production seen in DS by disrupting the IL-6 feedback loop. Another candidate is type I interferon blockade. Monoclonal antibodies against IFN-α or the type I IFN receptor (such as anifrolumab, approved for lupus) could be trialed in DS to tamp down the interferonopathy. However, given that IL-10 and other cytokines also contribute to DS immune phenotypes (8, 55, 73), single-cytokine targeting may be less effective than JAK inhibitors that cover multiple signals. Still, in patients with DS who have particular cytokine-driven complications (for example, TNF-α–mediated arthritis or IL-17–mediated psoriasis), using anti-TNF agents or IL-17 blockers could yield benefit. Small studies have documented that anti-TNF biologics can help DS patients with severe juvenile arthritis, highlighting the need to manage the inflammatory aspect of DS aggressively (19). Finally, therapies targeting the IL-1 family (like anakinra, an IL-1 receptor antagonist) might be relevant if IL-1 is found to significantly drive inflammation in a subset of DS individuals (basal IL-1α is elevated in many DS patients) (8, 74). Overall, a personalized medicine approach might be taken, measuring cytokine profiles in DS patients and selecting targeted inhibitors accordingly.

In addition to these targeted therapies, more general anti-inflammatory measures (e.g. statins, which have anti-inflammatory and interferon-modulating properties, or colchicine to dampen innate immune cell activation) could be considered as adjuncts. As research progresses, it will be important to evaluate the long-term safety of immune therapies in the DS population, who may have heightened infection risk. Encouragingly, early trials indicate that immune modulation (such as with JAK inhibitors) can be achieved without serious adverse effects in DS (3, 21, 66, 75). This suggests that immune activation in DS is, at least in part, reversible and amenable to therapeutic modulation (58). Dampening excessive inflammatory signaling may therefore alleviate autoimmune manifestations and, over longer time scales, could also influence neurodevelopmental or cognitive features that are shaped by chronic inflammation. Viewing DS through the lens of a systemic autoinflammatory disorder provides a clinical framework for addressing inflammation as a core component of the disease, rather than treating immune-related complications as isolated or secondary comorbidities.

## 6 Conclusion

DS has traditionally been viewed primarily as a neurodevelopmental condition; however, accumulating evidence indicates that trisomy 21 also has profound and sustained effects on immune system function. We note that Down syndrome encompasses both developmental and immune dysregulation components, and the autoinflammatory classification remains a working model rather than a definitive categorization. Multi-omics analyses, spanning transcriptomic, epigenomic, and immune profiling studies, consistently point to a shift toward innate immune activation, persistent interferon signaling, and enhanced expression of inflammatory gene programs. Together, these observations support the view that immune dysregulation is a central component of DS pathology, rather than a secondary consequence of developmental abnormalities. This perspective has important therapeutic implications. Targeting inflammatory and interferon-driven pathways addresses mechanisms that appear to be intrinsic to the disease and may therefore modify a broad range of clinical features. Continued integration of genomic, epigenetic, and immunological data will be essential for clarifying how gene dosage effects arising from trisomy 21 translate into aberrant immune responses. Such insights are likely to inform the rational design of clinical studies, including trials of interferon-directed therapies and other approaches aimed at restoring immune balance. Framing DS in this way links its developmental and immunological features within a shared pathogenic context and provides a basis for exploring immunomodulatory strategies to reduce its multisystem burden.
